# β-Adrenergic signaling blocks murine CD8^+^ T-cell metabolic reprogramming during activation: a mechanism for immunosuppression by adrenergic stress

**DOI:** 10.1007/s00262-018-2243-8

**Published:** 2018-09-18

**Authors:** Guanxi Qiao, Mark J. Bucsek, Nicolette M. Winder, Minhui Chen, Thejaswini Giridharan, Scott H. Olejniczak, Bonnie L. Hylander, Elizabeth A. Repasky

**Affiliations:** 1 Department of Immunology, Roswell Park Comprehensive Cancer Center, Elm and Carlton Streets, Buffalo, NY 14263 USA; 2 Present Address: Jacob School of Medicine and Biomedical Sciences, The State University of New York, University at Buffalo, 955 Main Street, Buffalo, NY 14203 USA

**Keywords:** Adrenergic signaling, CD8^+^ T-cell suppression, Metabolic reprogramming, Tumor immunology, T-cell activation, Glycolysis

## Abstract

Primary and secondary lymphoid organs are heavily innervated by the autonomic nervous system. Norepinephrine, the primary neurotransmitter secreted by post-ganglionic sympathetic neurons, binds to and activates β-adrenergic receptors expressed on the surface of immune cells and regulates the functions of these cells. While it is known that both activated and memory CD8^+^ T-cells primarily express the β2-adrenergic receptor (β2-AR) and that signaling through this receptor can inhibit CD8^+^ T-cell effector function, the mechanism(s) underlying this suppression is not understood. Under normal activation conditions, T-cells increase glucose uptake and undergo metabolic reprogramming. In this study, we show that treatment of murine CD8^+^ T-cells with the pan β-AR agonist isoproterenol (ISO) was associated with a reduced expression of glucose transporter 1 following activation, as well as decreased glucose uptake and glycolysis compared to CD8^+^ T-cells activated in the absence of ISO. The effect of ISO was specifically dependent upon β2-AR, since it was not seen in adrb2^−/−^ CD8^+^ T-cells and was blocked by the β-AR antagonist propranolol. In addition, we found that mitochondrial function in CD8^+^ T-cells was also impaired by β2-AR signaling. This study demonstrates that one mechanism by which β2-AR signaling can inhibit CD8^+^ T-cell activation is by suppressing the required metabolic reprogramming events which accompany activation of these immune cells and thus reveals a new mechanism by which adrenergic stress can suppress the effector activity of immune cells.

**Electronic supplementary material:**

The online version of this article (10.1007/s00262-018-2243-8) contains supplementary material, which is available to authorized users.

## Introduction

Immune organs are heavily innervated by the sympathetic nervous system (SNS) and immune cells express adrenergic receptors (ARs) for the SNS neurotransmitters norepinephrine (NE) and epinephrine (Epi). Physiological or psychological stressors which induce an SNS “fight or flight” response can rapidly modulate immune cell activity, supporting early observations in the field of psychoneuroimmunology which revealed that stressful events can negatively impact immune function and the onset and progression of disease [1]. For example, in an infectious disease model, SNS activity can suppress the capability of dendritic cells to activate an antiviral CD8^+^ T-cell response and this immunosuppression can be alleviated by treatment with a β2-AR antagonist (i.e., a β-blocker) [2]. However, many details regarding the intracellular pathways by which adrenergic signaling suppresses activation of CD8^+^ T-cells are still undefined.

More recently, it has also become clear that the SNS and stress-induced adrenergic signaling can play a key role in promoting tumor growth. We and several other groups have shown that tumor cells and associated stromal cells express adrenergic receptors and their signaling induces tumor cell proliferation and resistance to apoptosis, production of vascular endothelial growth factor (VEGF), epithelial–mesenchymal transition (EMT)-associated changes which facilitate metastasis, and induction of the angiogenic switch in endothelial cells [3–7].

Our group has become interested in identifying the mechanisms by which adrenergic stress affects immunity and has exploited a chronic, physiological adrenergic stress associated with housing temperature of mice. It is known that mice housed at IACUC mandated temperatures of ~ 22 °C are subjected to a mild, but chronic cold stress that has a profound impact on their physiology [8]. We discovered that the anti-tumor immune response is suppressed in these mice and that the level of circulating norepinephrine is significantly increased due to its role in stimulating heat production [4, 9]. When we housed mice at a thermoneutral temperature (~ 30 °C) instead of 22 °C, circulating norepinephrine levels significantly decreased and we observed that mice housed at 30 °C developed significantly stronger control of tumor growth and metastasis [8]. Later, we found that this immunosuppression is mediated by cold stress-induced release of NE and can be reversed by treating tumor-bearing mice with the pan-β-AR antagonist propranolol. We also found that blocking adrenergic signaling results in significantly improved tumor control and increased efficacy of the immune checkpoint inhibitor anti-PD-1 [10]. Moreover, our work has shown that the improved anti-tumoral effect of either housing mice at thermoneutrality or inhibition of β-AR signaling by β-AR blockers is dependent on CD8^+^ T-cells. Because the β2-adrenergic receptor (β2-AR) is the primary subtype expressed on immune cells (including T-cells, dendritic cells, B cells, and macrophages [11, 12]), we further investigated the role of this receptor using an adrb2^−/−^ mouse. Our results supported the conclusion that the immunosuppressive effects of NE are mediated through the β2-AR [10]. Other studies have also confirmed that activated and memory CD8^+^ T-cells express β2-ARs, and their activation and function are impaired by β2-AR signaling [13].

TCR-induced activation is known to drive T-cells to undergo metabolic reprogramming (increasing glycolysis and oxidative phosphorylation) to meet the cellular energetic and biosynthetic demands of activation, differentiation, and effector function [14–17]. Previously, we have demonstrated that effector CD8^+^ T-cells isolated from tumors of mice housed at 22 °C have lower surface expression of the glucose transporter GLUT1 than do CD8^+^ T-cells isolated from mice housed at thermoneutrality 30 °C [8]. Here, we demonstrate that β2-AR stimulation suppresses metabolic reprogramming as judged by both glycolysis and oxidative phosphorylation and could, therefore, be a major mechanism by which adrenergic stress suppresses cellular immune activity in the setting of anti-tumor immunity, or other T-cell-dependent immune functions.

## Materials and methods

### Isolation and culture of CD8^+^ T-cells

8–12-week-old female BALB/cAnNcr (BALB/c), C57BL/6NCr (C57BL/6), and adrb2^−/−^ mice on a BALB/c background [13] were used for isolating CD8^+^ T-cells. Mice were sacrificed and spleens and lymph nodes were collected. Single-cell suspensions were made from spleen and lymph nodes by crushing and filtering through a 70 µm nylon cell strainer (Corning). Red blood cells were lysed by ACK lysing buffer (Gibco). CD8^+^ T-cells were purified from suspensions using a negative isolation kit (CD8a^+^ T-cell isolation kit, Miltenyi Biotec) following the manufacturer’s protocols. CD8^+^ T-cells were cultured at 1 × 10^6^/ml in the presence of plate-coated anti-CD3/anti-CD28 antibodies (anti-CD3 clone 145-2C11 2 µg/ml; anti-CD28 clone 37.51 2 µg/ml, BioXcell) in RPMI 1640 (Corning) supplemented with 10% fetal bovine serum, 1% l-glutamine, 1% Penicillin/Streptomycin, 0.1% HEPES buffer, 0.1% sodium pyruvate, 0.05% 2-mercaptoethanol, and 0.1% non-essential amino acids. CD8^+^ T-cells were activated in the presence or the absence of 10 µM isoproterenol in PBS. OT-1 spleen cell suspensions were cultured at 2 × 10^6^/ml with the OVA peptide (SIINFEKL, 10 nM) in supplemented media as indicated above.

### Metabolic assays

For all extracellular flux assays, CD8^+^ T-cells were plated on cell-tak-coated Seahorse XF96 cell-culture microplates at a density of 2 × 10^5^ cells per well. The assay plates were spin seeded for 5 min at 1000 rpm and incubated at 37 °C without CO_2_ prior to performing the assay on the Seahorse Bioscience XFe96. The mitochondrial stress test was performed in XF Base Media containing 10 mM glucose, 1 mM sodium pyruvate, and 2 mM l-glutamine and the following inhibitors were added at the final concentrations: oligomycin (2 µM), carbonyl cyanide 4-(trifluoromethoxy) phenylhydrazone (FCCP) (2 µM), and rotenone/antimycin A (0.5 µM each). The glycolytic stress test was performed in XF Base Media containing 2 mM l-glutamine and the following reagents were added at the final concentrations: glucose (10 mM), oligomycin (2 µM), and 2-deoxy-d-glucose (2-DG, 50 mM).

### Flow cytometry

Cells were harvested at either 24 h or 48 h after activation and washed twice with flow running buffer (0.1% BSA in PBS). Cells were stained with following antibodies: anti-CD3 (clone 145-2C11) conjugated to either APC-Cy7, BV786, or PE; anti-CD8α (clone 53–6.7) conjugated to BUV395, APC, or FITC; anti-CD69 (clone H1.2F3) conjugated to PE; anti-CD44 (clone IM7) conjugated to PE; and anti-CD62L (clone MEL-14) conjugated to APC. All antibodies were purchased from BD biosciences. Live/dead fixable violet, aqua, and yellow cell dyes from Thermo Fisher were used to gate out dead cells.

For β2-adrenergic receptor surface staining, cell-surface markers were first labeled and live/dead fixable dye was used to gate out dead cells, and then, cells were incubated with β2-AR antibody (clone H-20), following secondary antibody conjugated with Ax647.

For intracellular staining, cell-surface markers were first labeled and live/dead fixable dye was used to gate out dead cells, and then, cells were fixed and permeabilization using the FoxP3/transcription factor staining buffer set (Thermo Fisher) following the manufacturer’s protocol. Cells were then stained with either anti-GLUT1/Ax647 (clone EPR3915) or isotype control/Ax647 from Abcam.

Glucose uptake was analyzed using 2-NBDG {2-[*N*-(7-nitrobenz-2-oxa-1,3-diazol-4-yl) amino]-2-deoxyglucose}, a fluorescent glucose analog. Cells were incubated in glucose-free RPMI1640 with 100 µM 2-NBDG (Sigma) at 37 °C for 30 min and then stained with extracellular antibodies and live/dead fixable dye as previous described.

For staining with mitochondrial dye, cells were first stained with extracellular antibodies and live/dead fixable dye as previous described and then incubated with RPMI 1640 containing either 30 mM MitoTracker Green FM (mitochondrial mass) or MitoTracker Orange CMTMRos (mitochondrial membrane potential) at 37 °C for 30 min. All mitochondria dyes were purchased from Thermo Fisher.

All flow data were collected using an LSR Fortessa flow cytometry (BD biosciences) and analyzed with FlowJo v10.

### ImageStream

Cells were harvested at 48 h after activation and intracellular GLUT1 staining was performed. Data were collected using ImageStream X Mark II (Amnis, MilliporeSigma).

### Statistical analysis

Data between two groups were compared using the Student’s two-tailed *t* test. Data between multiple groups, one-way ANOVA with Tukey adjusted post-hoc tests. All data are graphed as mean ± SEM.

## Results

### β-Adrenergic receptor signaling inhibits glucose transporter expression during CD8^+^ T-cell activation

**Fig. 1:**
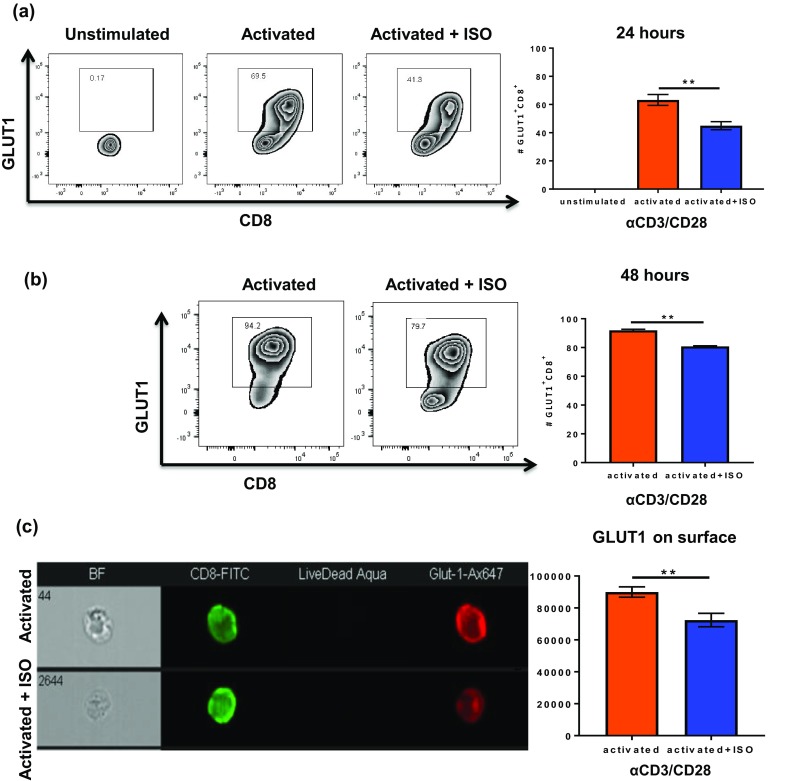
βAR signaling inhibits glucose transporter 1 (GLUT1) up-regulation during T-cell activation. CD8^+^ T-cells from BALB/c mice were isolated and purified from lymph node and spleen of non-tumor-bearing mice, and activated with anti-CD3/CD28 antibodies with or without isoproterenol (ISO). GLUT1 expression was tested by flow cytometry. GLUT1 expression in CD8^+^ T-cells; **a** at 24 h; **b** at 48 h after activation; **c** GLUT1 surface expression was tested by imageStream; *n* = 4–6; data were analyzed using Student’s *t* test, **p < 0.01

Previously, we reported that reducing adrenergic stress by housing mice at thermoneutrality (30 °C) compared to 22 °C resulted in increased GLUT1 expression during activation [8]. Here, we first asked whether adrenergic suppression of GLUT1 expression could be reversed by treating tumor-bearing mice with the β-blocker propranolol. As shown in Supplementary Fig. 1, in a melanoma model (B16-OVA), tumor-infiltrating CD8^+^ T-cells isolated from tumors of mice housed at 22 °C and treated with β-blockers do express higher levels of GLUT1 than cells from control mice receiving PBS. Therefore, we hypothesized that β-AR signaling suppresses CD8^+^ T-cell effector function by suppressing GLUT1 expression, thereby inhibiting metabolic reprogramming during activation. To investigate this hypothesis, we examined the effects of adrenergic signaling on CD8^+^ T-cells activated in the presence of the β-AR agonist isoproterenol (ISO). CD8^+^ T-cells were isolated from spleen and lymph nodes from BALB/c mice and activated with plate-bound anti-CD3/CD28 antibodies in the presence or the absence of ISO and GLUT1 expression was measured by flow cytometry (Fig. 1). It has been reported that GLUT1 expression can be detected at 24 h after activation [18, 19]; therefore, GLUT1 expression was tested both at 24 h and 48 h after activation. GLUT1 expression was undetectable by flow cytometry in unstimulated CD8^+^ T-cells (Fig. 1a). GLUT1 expression in control and ISO-treated CD8^+^ T-cells was examined (Fig. 1a, b) after activation. Comparison showed that adrenergic signaling significantly reduced GLUT1 expression in CD8^+^ T-cells during activation. During T-cell activation, GLUT1 expression is increased and it is translocated to the cell membrane to take up glucose from the outside environment [18]. To determine whether the decreased expression of GLUT1 that was observed by flow cytometry represented decreased cytoplasmic and/or cell-surface GLUT1, the GLUT1 expression was localized using the ImageStream. Our results showed that adrenergic signaling decreased GLUT1 cell-surface expression (Fig. 1c). By treating CD8^+^ T-cells with different doses of ISO, we were able to demonstrate that the effect of ISO on GLUT1 expression is dose dependent (Supplementary Fig. 2a) without affecting cell viability. In addition, the effect of ISO can be blocked by the β-AR antagonist propranolol (Supplementary Fig. 2b) and our results showed that propranolol itself did not have an effect on GLUT1 expression. However, the effect of ISO is not reversible by merely washing it out (Supplementary Fig. 2c), which indicates that the effect of ISO is on the initiation, or at least an early stage, of T-cell activation. Adrenergic signaling also suppressed GLUT1 expression in a second strain of mice, C57BL/6 (Supplementary Fig. 3).

### β-AR signaling inhibits glucose transporter expression during CD8^+^ T-cell activation primarily through β2-AR and β2-AR expression is associated with CD28 co-stimulation

**Fig. 2:**
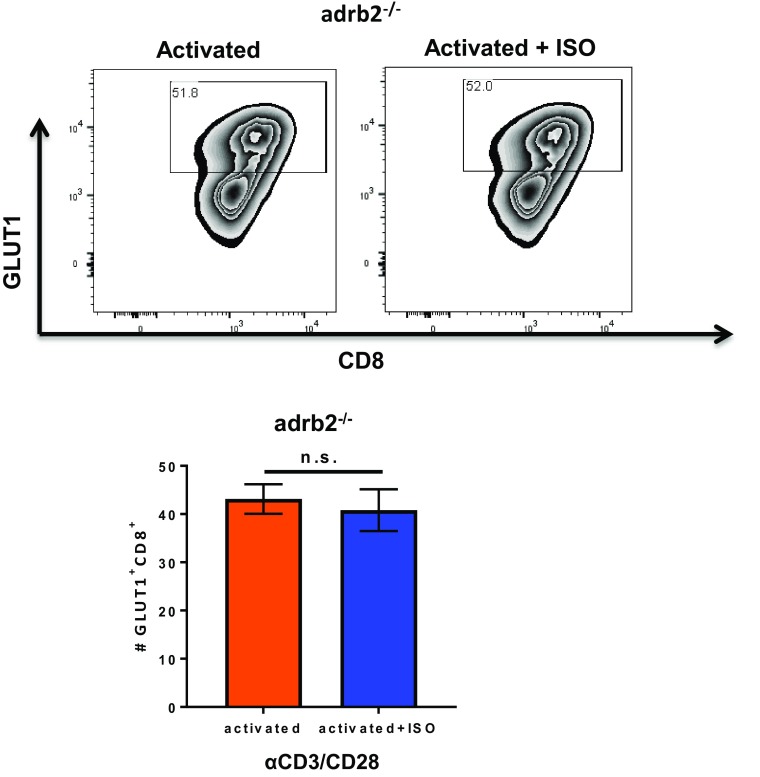
Inhibition of GLUT1 up-regulation by adrenergic signaling depends on the β2-AR. CD8^+^ T-cells were isolated and purified from spleen and lymph nodes of adrb2^−/−^ mice. Cells activated with anti-CD3/CD28 antibodies with or without ISO showed no difference in expression of GLUT1 as quantified by flow cytometric analysis; *n* = 4; data were analyzed using Student’s *t* test

Studies have shown that immune cells primarily express β2-AR and the effect of catecholamines on CD8^+^ T-cells is through β2-AR [13]. First, we examined the β2-AR expression on CD8^+^ T-cells and found that β2-AR is undetectable by flow cytometry in unstimulated CD8^+^ T-cells, but when CD8^+^ T-cells were stimulated with anti-CD3/CD28 antibodies, the expression of β2-AR was increased. However, when CD8^+^ T-cells were stimulated with anti-CD3 antibody alone, β2-AR expression was undetectable as in unstimulated T-cells, which indicates that the increased expression of β2-AR during T-cell activation with anti-CD3/CD28 antibodies is associated with CD28 co-stimulation (Supplemental Fig. 4). To test the hypothesis that the inhibitory effect of adrenergic signaling on GLUT1 expression is specifically through β2-AR, we investigated the response of CD8^+^ T-cells from BALB/c adrb2^−/−^ mice. These knockout mice have been used previously in several other studies and both T and B cells from these mice have been activated in vitro. From these studies, there appear to be no obvious differences between T and B cells from the wild-type mice and adrb2^−/−^ mice [13, 20, 21]. At baseline, these mice do not show major defects. However, we find that differences between wild-type and adrb2^−/−^ mice appear when the animals are stressed [10, 22]. In cells lacking β2-AR, there was no difference in GLUT1 expression during activation between the control and ISO-treated cells (Fig. 2), indicating that β2-AR is the main receptor responsible for the decreased up-regulation of glucose transporter during CD8^+^ T-cell activation. In addition, we activated and compared CD8^+^ T-cells from wild-type and knockout mice, and in the absence of ISO, there were no differences in GLUT1, CD69, and CD44 expressions (Supplemental Fig. 5), which confirmed the previous study that found no obvious difference between activation of wild-type and adrb2^−/−^ CD8^+^ T-cells.

### β-AR signaling inhibits uptake of glucose during CD8^+^ T-cell activation

**Fig. 3:**
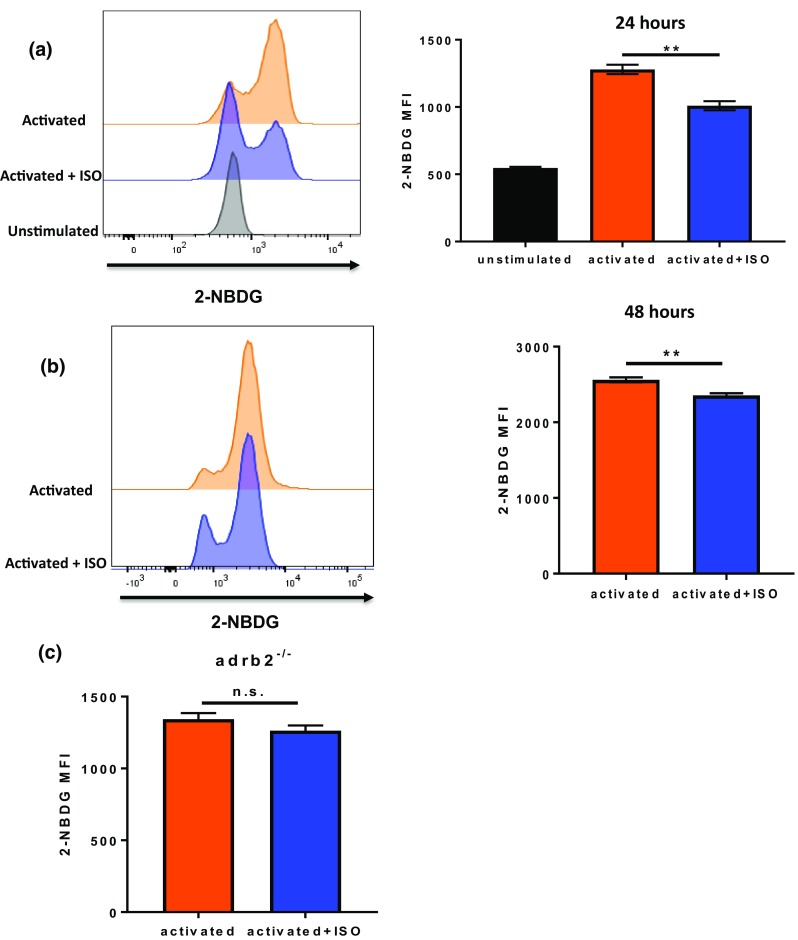
Adrenergic signaling inhibits glucose uptake during CD8^+^ T-cell activation. CD8^+^ T-cells from BALB/c mice were isolated and purified from lymph node and spleen and activated with anti-CD3/CD28 antibodies with or without ISO. Uptake of the non-metabolized glucose analog 2-NBDG was analyzed by flow cytometry. Glucose uptake by CD8^+^ T-cells **a** at 24 h; **b** at 48 h after activation; and **c** by CD8^+^ T-cells from adrb2^−/−^ mice; *n* = 4–6; data were analyzed using Student’s *t* test, **p < 0.01

Whether reduced expression of GLUT1 led to diminished uptake of glucose was determined by incubating activated CD8^+^ T-cells with 2-NBDG, a fluorescent glucose analog which is transported into cells, but cannot be further metabolized, allowing it to be quantitatively measured by flow cytometry. As expected, unstimulated CD8^+^ T-cells had low glucose uptake (Fig. 3a). By comparison, at both 24 h and 48 h after activation, ISO-treated CD8^+^ T-cells took up less glucose than non-treated CD8^+^ T-cells (Fig. 3a, b). This inhibition of GLUT1 expression and glucose uptake by β-AR was also found in CD8^+^ T-cells from C57BL/6 mice (Supplementary Fig. 6a). In contrast, adrenergic signaling did not impair glucose uptake in CD8^+^ T-cells from adrb2^−/−^ mice (Fig. 3c).

Splenocytes from OT-1 mice were used to test whether the effect of β2-AR is restricted to strong activation through anti-CD3/CD28 antibodies. Our results showed that even through all cells express GLUT1, the glucose uptake was decreased by β-AR signaling (Supplementary Fig. 6b). In addition, other published studies showing that β2-AR suppresses TCR-mediated CD8^+^ T-cell effector function also showed that OT-1 effector function is inhibited by β-AR signaling [13]. Overall, these data strongly suggest that the suppressive effects of β2-AR signaling are not restricted to cells which are activated by anti-CD3/CD28 antibodies.

### β-AR signaling during CD8^+^ T-cell activation inhibits glycolysis

**Fig. 4:**
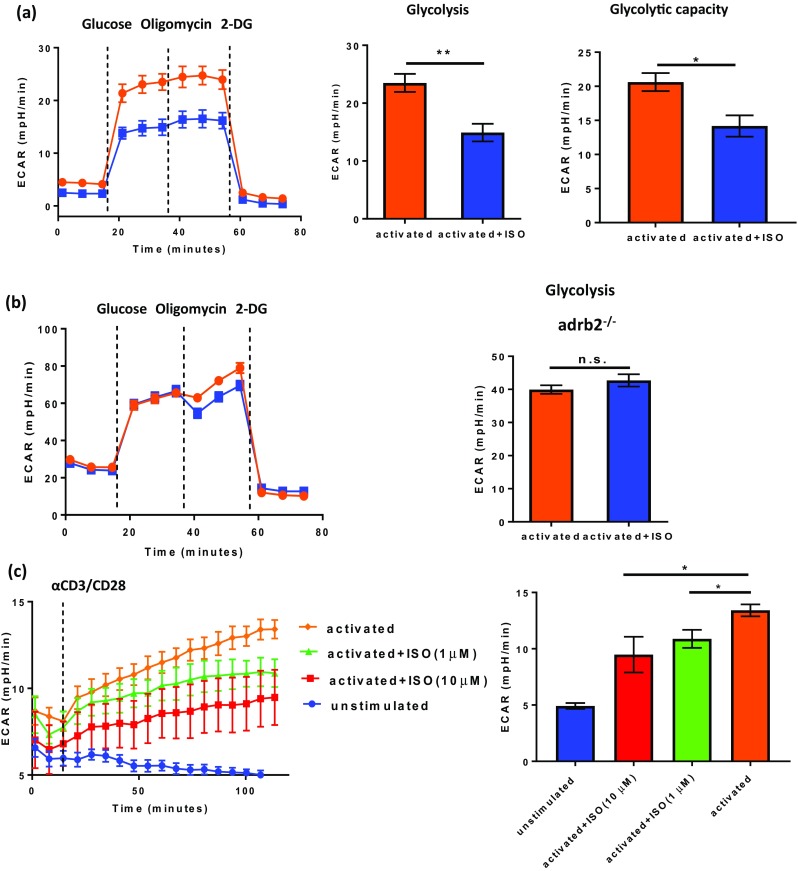
β2-AR signaling during T-cell activation inhibits glycolysis in a dose-dependent manner. CD8^+^ T-cells from BALB/c (**a**) or adrb2^−/−^ mice (**b**) were isolated and purified from lymph node and spleen and activated with anti-CD3/CD28 antibodies with or without ISO for 48 h. Glycolysis was tested with a Seahorse Extracellular Flux Analyzer [addition of reagents indicated by dotted lines: (1) glucose; (2) oligomycin; and (3) 2-DG]; *n* = 4; data were analyzed using Student’s *t* test, *p < 0.05, **p < 0.01. **c** Real-time analysis of in situ-activated CD8^+^ T-cells; bar graph compares the final values at 120 min. *n* = 4. Dotted line indicated timepoint when anti-CD3/CD28 antibodies were added to activate the T-cells

Up-regulation of glycolysis during metabolic reprogramming is dependent upon increased glucose uptake [18, 19] and our data show that GLUT1 and glucose uptake are inhibited by β-AR signaling. To determine how this impacts glycolysis, we used the Seahorse Extracellular Flux Analyzer to measure glycolysis in CD8^+^ T-cells (controls and ISO treated) following 48 h of activation. The results of the glycolytic stress test (represented by changes in the extracellular acidification rate, ECAR) showed that the expected increase in glycolysis following activation of naïve CD8^+^ T-cells, as well as glycolytic capacity, was impaired by ISO (Fig. 4a). A second measure of CD8^+^ T-cell activation, increased CD69 expression, was also significantly reduced at both 24 h and 48 h after activation in the presence of ISO (Supplementary Fig. 7a, b). In addition, CD44 expression was decreased and there were fewer effector memory (CD44^high^ CD62L^low^) CD8^+^ T-cells (Supplementary Fig. 7c, d). We also found that CD28 was decreased at 48 h in the presence of ISO (Supplementary Fig. 7e). In addition to the above experiments with T-cells from BALB/c mice, the inhibition of glycolytic function by β-AR signaling was also found in CD8^+^ T-cells from C57BL/6 mice (Supplementary Fig. 8). In the absence of β2-AR, there was no difference in glycolysis between control and ISO-treated activated CD8^+^ T-cells (Fig. 4b).

Next, we activated CD8^+^ T-cells in situ (in the Seahorse) to detect T-cell activation responses and ECAR was measured in real time to investigate effects of adrenergic signaling at early timepoints. We again found that glycolysis was increased during activation of CD8^+^ T-cells; however, the addition of ISO decreased glycolysis in a dose-dependent manner even at these early stages of activation (Fig. 4c).

### β-AR signaling impairs CD8^+^ T-cell mitochondrial function and mass increase during activation

**Fig. 5:**
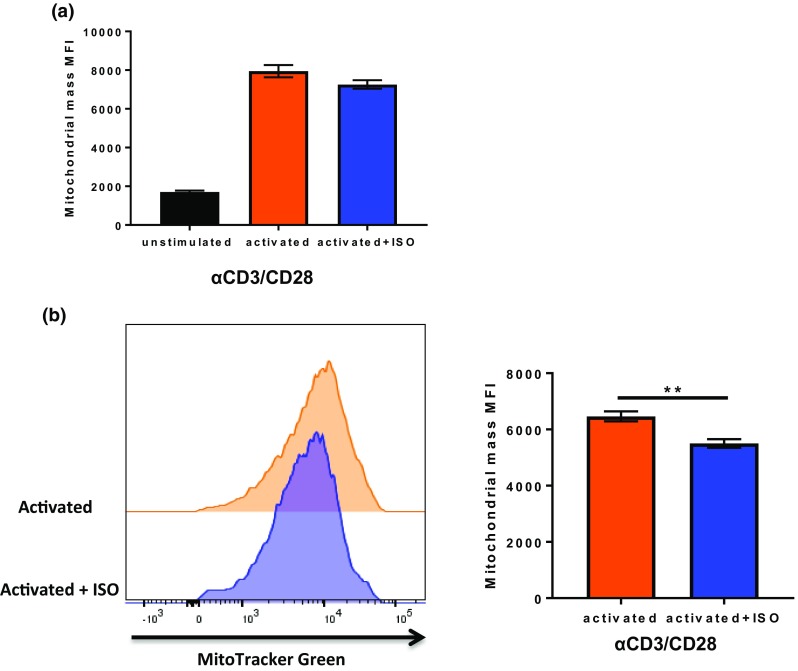
β2-AR signaling inhibits mitochondrial mass increase during T-cell activation. CD8^+^ T-cells from BALB/c mice were isolated and purified from lymph node and spleen and activated with anti-CD3/CD28 antibodies with or without ISO. Mitochondrial mass was determined by staining with mitoTracker Green FM and quantified by flow cytometry. CD8^+^ T-cell mitochondrial mass **a** at 24 h; **b** at 48 h after activation; *n* = 4; data were analyzed using Student’s *t* test, **p < 0.01

**Fig. 6:**
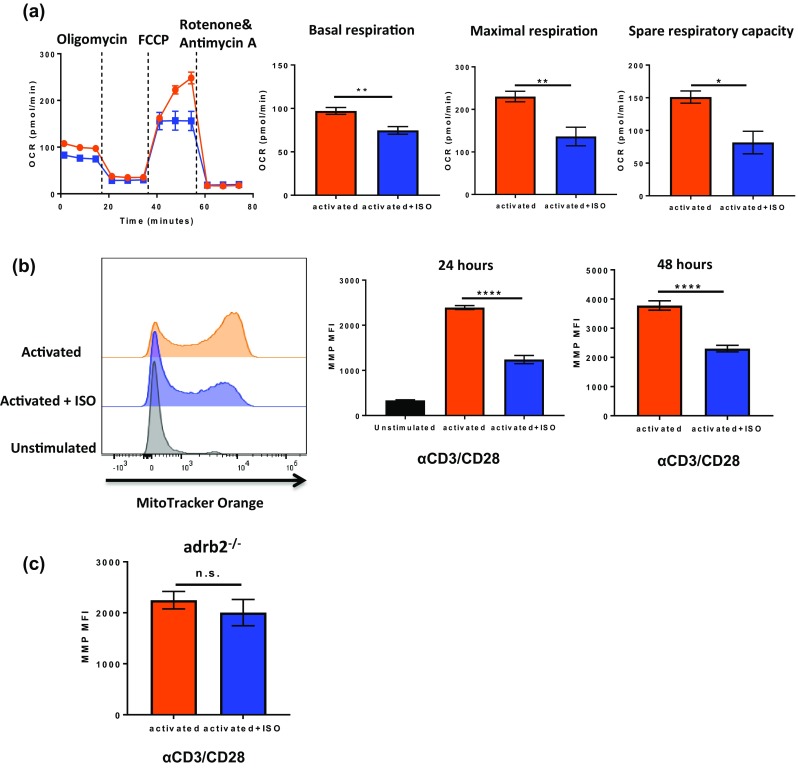
β2-AR signaling inhibits mitochondrial respiration during T-cell activation. CD8^+^ T-cells from BALB/c mice were isolated and purified from lymph node and spleen activated with anti-CD3/CD28 antibodies with or without ISO. **a** Mitochondrial respiration was tested using Seahorse Extracellular Flux Analyzer [addition of reagents indicated by dotted lines: (1) oligomycin; (2) FCCP; and (3) antimycin A and rotenone] *n* = 6; **b** mitochondrial membrane potential was tested by flow cytometry at 24 h and 48 h. *n* = 4. **c** Mitochondrial membrane potential in CD8^+^ T-cells from adrb2^−/−^ mice; *n* = 4; data were analyzed using Student’s *t* test, *p < 0.05, **p < 0.01, ****p < 0.0001

T-cells increase aerobic glycolysis to support their activation, differentiation, and proliferation. However, the mitochondria remain a vital part of T-cell metabolism, since effector T-cells also significantly up-regulate mitochondrial oxidative activity [15]. To investigate the effects of adrenergic signaling on mitochondria, mitochondrial mass was measured by incubating activated CD8^+ ^ T-cells +/- ISO with Mitotracker Green FM. We found that at 24 h, there was a significant increase in mitochondrial mass in both groups compared to unstimulated cells, but slightly less in the ISO-treated group (Fig. 5a); this difference was significant at 48 h (Fig. 5b). Following this observation, we performed a mitochondrial stress test using the Seahorse Extracellular Flux Analyzer to compare mitochondrial respiration (represented by oxygen consumption rate, OCR) between non-treated and ISO-treated CD8^+^ T-cells. The results showed that basal mitochondrial respiration was slightly less in ISO-treated CD8^+^ T-cells (Fig. 6a). However, the maximum mitochondrial respiration rate and mitochondrial spare respiratory capacity (SRC) were significantly decreased in ISO-treated CD8^+^ T-cells (Fig. 6a), which may indicate mitochondrial dysfunction and a defect in metabolic fitness [16]. The inhibition of mitochondrial respiration by β-AR signaling was also found using CD8^+^ T-cells from C57BL/6 mice (Supplementary Fig. 9). These data demonstrate that β-AR signaling impairs mitochondrial respiration in CD8^+^ T-cells during activation. Since we observed a decrease in mitochondrial respiration rate, we further investigated mitochondrial function by measuring the mitochondrial membrane potential (MMP). Correlating with the reduced mitochondrial respiration rate, we found that there was a decrease in mitochondrial membrane potential (without difference in cell death) in the ISO-treated CD8^+^ T-cells compared to that of the non-treated CD8^+^ T-cells at both 24 and 48 h after activation (Fig. 6b). To confirm that the impairment of MMP by ISO is mainly through β2-adrenergic receptor signaling, adrb2^−/−^ CD8^+^ T-cells were tested and there was no effect on MMP in these cells (Fig. 6c). Altogether, these data suggest that the inhibition of mitochondrial respiration by β-AR signaling may cause mitochondrial dysfunction in CD8^+^ T-cell during activation.

## Discussion

Improving the anti-tumor immune response is a major goal of cancer research. Much of this effort is focused on improving CD8^+^ T-cell effector activity. These effector responses are suppressed by a variety of immune escape mechanisms and it is critical to identify additional immunosuppressive mechanisms that can be targeted to improve immunity against cancers and other diseases. Recent research in immunology highlights how changes in cell metabolism support immune cell activation, growth, proliferation, and effector function and eventually, a return to homeostasis. During these changes, glucose metabolism is vital in regulating T-cell activation, differentiation, cytokine production, cytolytic function, and even the onset of cell death [14, 23–27]. Naïve resting T-cells have a relatively low metabolic demand and predominately metabolize glucose through oxidative phosphorylation. During T-cell activation, signaling by the T-cell receptor, co-stimulatory molecules, and cytokines drive increased T-cell metabolism predominately through induction of “metabolic reprogramming” in which T-cells become dependent on glycolysis, although OXPHOS persists and also increases [15]. This high level of glycolytic flux in activated T-cells is vital to their effector function [15, 28]. Withdrawal of antigenic signal or nutrients such as glucose can induce metabolic stress in T-cells which impairs their effector function and can result in apoptosis [29, 30]. In addition, evidence shows that anergic T-cells are metabolically anergic, and failure to up-regulate metabolic pathways upon T-cell activation leads to a hypo-responsive phenotype [31]. Therefore, having a sufficient glucose supply and proper regulation of metabolism is critical for successful T-cell activation and effector function.

Here, we have demonstrated that β-AR signaling inhibits CD8^+^ T-cell metabolic reprogramming during activation. To meet the high demand for glucose, activated T-cells usually increase glucose transporter expression (mainly GLUT1) and/or surface trafficking [28, 32, 33]. By activating CD8^+^ T-cells with anti-CD3/CD28 antibodies in the presence or the absence of the β-AR agonist isoproterenol, we found that adrenergic signaling impairs GLUT1 expression, glucose uptake, and glycolysis, primarily through β2-AR signaling. Mitochondrial respiration was also seen to be inhibited, particularly spare respiratory capacity, which is considered a measure of metabolic fitness. Finally, we found that β-adrenergic signaling decreases mitochondrial mass and mitochondrial membrane potential, suggesting an overall impairment of mitochondrial function.

Although these differences that we observed in activation-associated metabolic changes are subtle, they are robust in replication and statistical significance. However, it is important to recognize that stress signaling occurs frequently in physiological settings; therefore, one would not expect major effects from mild chronic stressors (such as housing temperature), or otherwise, immune response would be seriously impaired by repeated mild stress and this is generally not the case. Our thinking is that in the setting of chronic stress, T-cell activation occurs, but does not reach its full potential, and thus over time, this subtle immunosuppression could result in impaired immunosurveillance, or impaired anti-tumor immunity, which is in line with our previous finding that chronic stress-induced adrenergic signaling impairs anti-tumor immunity and that blocking adrenergic signaling over time results in significantly improved tumor growth control.

Several conditions of the TME are known to compromise T-cell effector function. Low availability of glucose, glutamine, and amino acids in the tumor microenvironment inhibits T-cell function, while metabolites such as lactic acid are immunosuppressive. Now, our data suggests the possibility that increased metabolic demand due to adaptive thermogenesis, or other metabolic outcomes of adrenergic stress, may further deplete available resources and we speculate that this generates, through β-AR signaling, a diminished capacity of CD8^+^ T-cells to acquire sufficient nutrients. Our work suggests the possibility that stress may suppress the metabolically expensive immune response to direct limited resources to more immediately beneficial survival functions.

There are still many questions that need to be addressed. The precise pathways through which β-adrenergic signaling inhibits metabolic reprogramming are still unknown. There is research showing that GLUT1 and glucose uptake are increased through the CD28–PI3k–Akt–mTOR pathway [19] and the decrease in CD28 expression which we observed in the presence of a β-adrenergic agonist suggests that adrenergic signaling may inhibit this signal pathway. Our ongoing research is assessing in more detail the role of this pathway in regulating the impact of adrenergic stress on T-cell metabolism. Furthermore, the potential effects of β-adrenergic signaling on glucose flux through various metabolic pathways are unknown and could provide further insights into regulation of T-cell metabolism. Another important direction will be to determine if the effects of β-adrenergic signaling on metabolism that we found in vitro also occur in the tumor microenvironment. While ligation of the TCR and co-stimulatory receptors drives metabolic reprogramming, ligation of coinhibitory receptors such as PD-1 has the opposite effect, decreasing glycolysis and mitochondrial function [34]. Therefore, it will be important to investigate how adrenergic signaling affects these coinhibitory receptors.

In addition, there is evidence showing that β-AR signaling also regulates Th1/Th2 differentiation of CD4^+^ T-cells, polarizing them towards a Th2 phenotype [35, 36]. Based on our work, it is likely that β-AR signaling regulates differentiation of CD4^+^ T-cells by altering metabolism, but this remains to be investigated. It will also be important to see how adrenergic signaling alters the metabolism of immunosuppressive cells. In light of these findings, it is likely that the anti-tumor efficacy of CD8^+^ T-cells could be increased by β-blockers; perhaps, as clinical trials testing the repurposing of β-blockers in combination with other therapies such as chemotherapy or immune checkpoint inhibitors progress, these questions can be addressed by analysis of patient specimens in addition to preclinical mouse models.

## Electronic supplementary material

Below is the link to the electronic supplementary material.

Supplementary material 1 (PDF 261 KB)

## Abbreviations

β-ARs: Beta-adrenergic receptors
2-DG: 2-Deoxy-d-glucose
ECAR: Extracellular acidification rate
EMT: Epithelial–mesenchymal transition
Epi: Epinephrine
FCCP: Carbonyl cyanide 4-(trifluoromethoxy) phenylhydrazone
GLUT1: Glucose transporter 1
IACUC: The Institutional Animal Care and Use Committee
ISO: Isoproterenol
MMP: Mitochondrial membrane potential
2-NBDG: 2-(*N*-(7-Nitrobenz-2-oxa-1,3-diazol-4-yl) amino)-2-deoxyglucose
NE: Norepinephrine
OCR: Oxygen consumption rate
SNS: Sympathetic nervous system
TCR: T-cell receptor
TME: Tumor microenvironment
VEGF: Vascular endothelial growth factor
