# Supplementary material for: β-Adrenergic signaling blocks murine CD8+ T-cell metabolic reprogramming during activation: a mechanism for immunosuppression by adrenergic stress
Source: Cancer Immunol Immunother. 2018 Sep 18;68(1):11–22. doi: 10.1007/s00262-018-2243-8 (PMC6326964; doi:10.1007/s00262-018-2243-8)
Supplement: Supplementary file 1 — Supplementary material 1 (PDF 261 KB) [file 262_2018_2243_MOESM1_ESM.pdf]

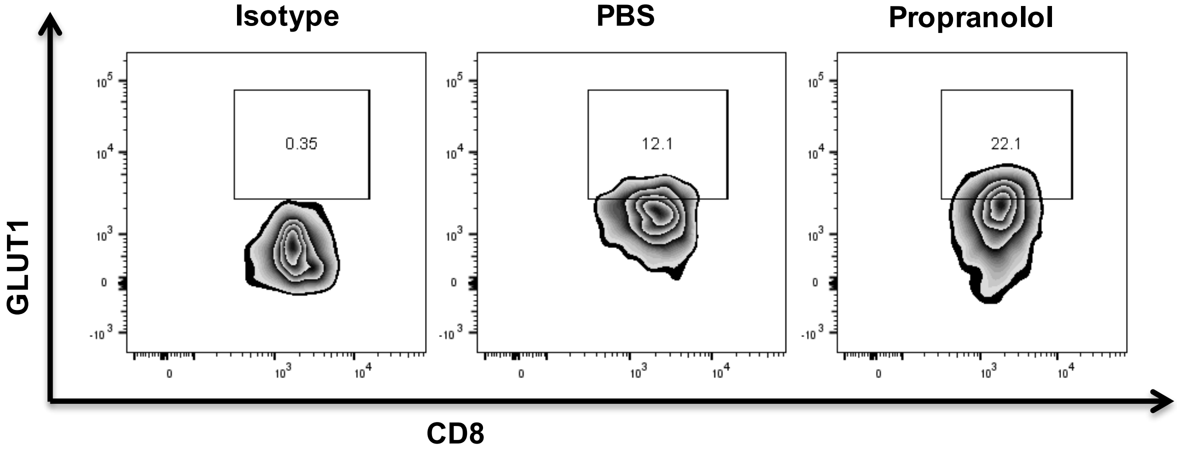

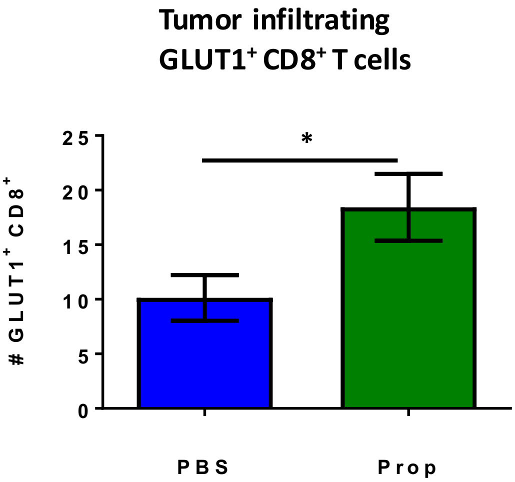

Supplemental Figure 1. GLUT1 expression is increased in tumor infiltrating CD8⁺ T-cells from mice treated with the β-adrenergic receptor antagonist propranolol

2×10^5 B16-OVA cells were injected into C57BL/6 mice and tumor growth was monitored. Mice were treated with either PBS or the β-adrenergic receptor antagonist propranolol. At day 28, tumor infiltrating $CD8^+$ T-cells were isolated and GLUT1 expression quantified by flow cytometry. N=10; Data was analyzed using Student's t test , *$p<0.05$

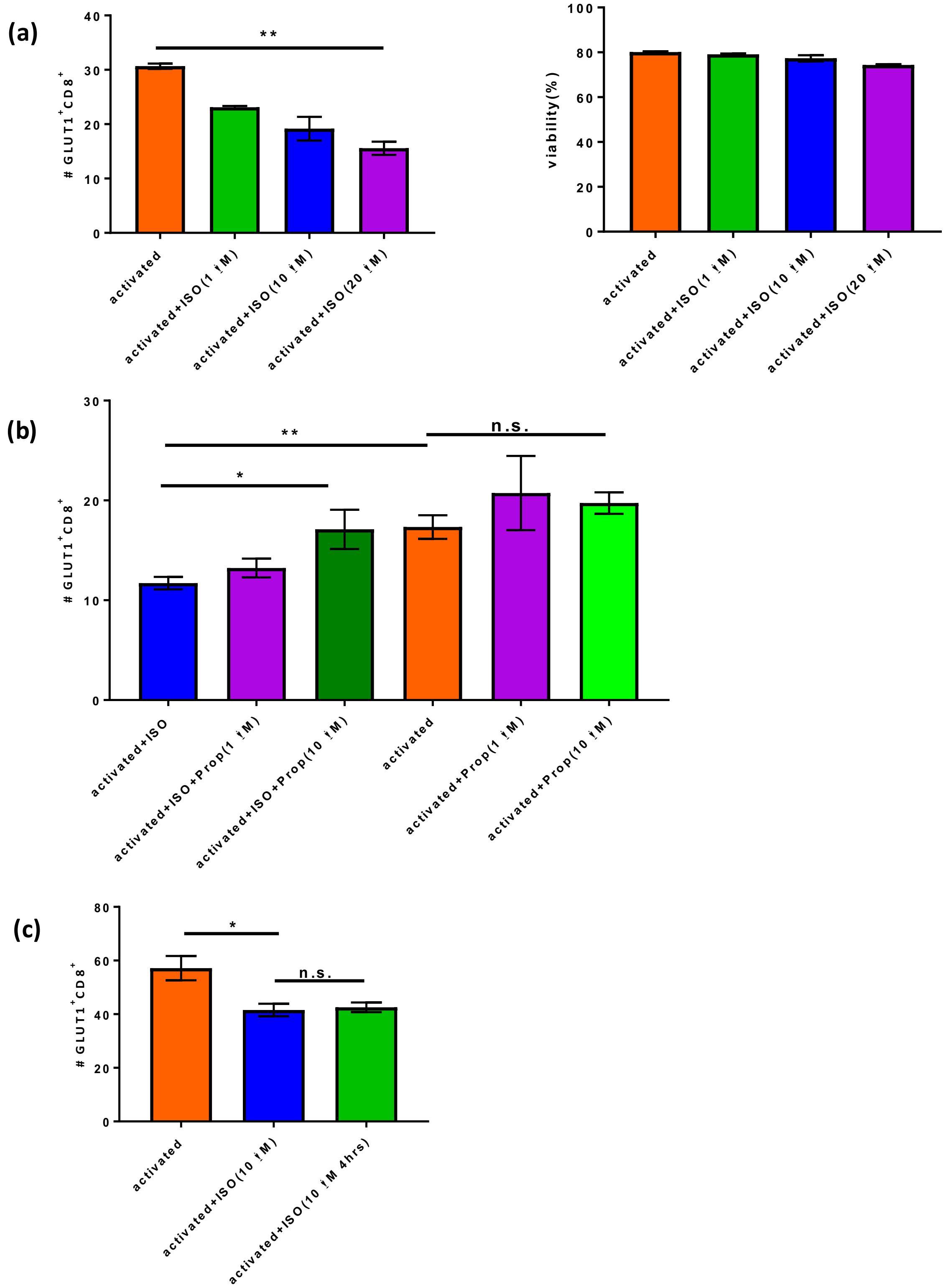

Supplemental Figure 2. β-AR signaling inhibits GLUT1 up-regulation during T cell activation in a dose dependent manner and can be blocked by β-AR antagonist Propranolol CD8+ T-cells from BALB/c mice were isolated and purified from lymph node and spleen of non-tumor-bearing mice, and activated with anti-CD3/CD28 antibodies with or without isoproterenol. GLUT1 expression was tested by flow cytometry. GLUT1 expression in CD8+ T-cells **(a)** different does (1, 10, 20μM) of isoproterenol **(b)** isoproterenol+/-propranolol **(c)** CD8+ T-cells were activated with or without isoproterenol. After 4 hours, isoproterenol was washed out . n=3-4; Data was analyzed using Student's t test , *$p<0.05$, **$p<0.01$

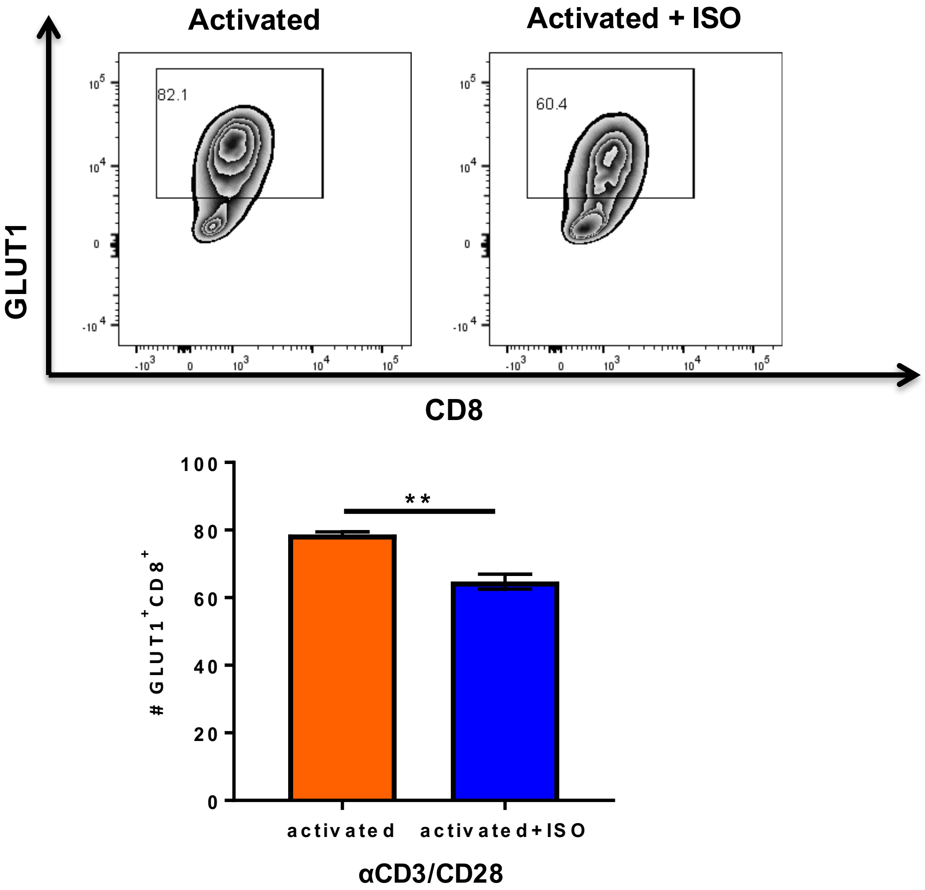

Supplemental Figure 3. β-AR signaling inhibits GLUT1 up-regulation during T cell activation of CD8+ T-cells from C57BL/6 mice CD8+ T-cells were isolated and activated in the presence or absence of ISO. Expression of GLUT-1 was tested at 48 hours after activation. n=6; Data was analyzed using Student's t test , **p<0.01

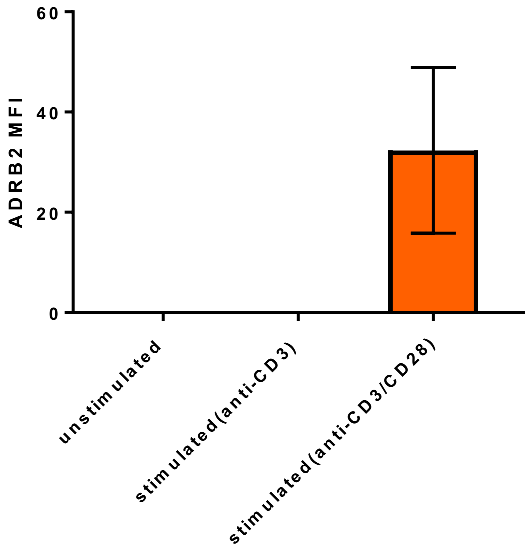

Supplemental Figure 4. β2-adrenergic receptor (ADRB2) expression is increased after activation and is associated with CD28 co-stimulation $CD8^+$ T-cells were isolated and activated with either anti-CD3 or anti-CD3/CD28 antibodies. β2-adrenergic receptor was measured by flow cytometry. n=3; Data was analyzed using Student's t test.

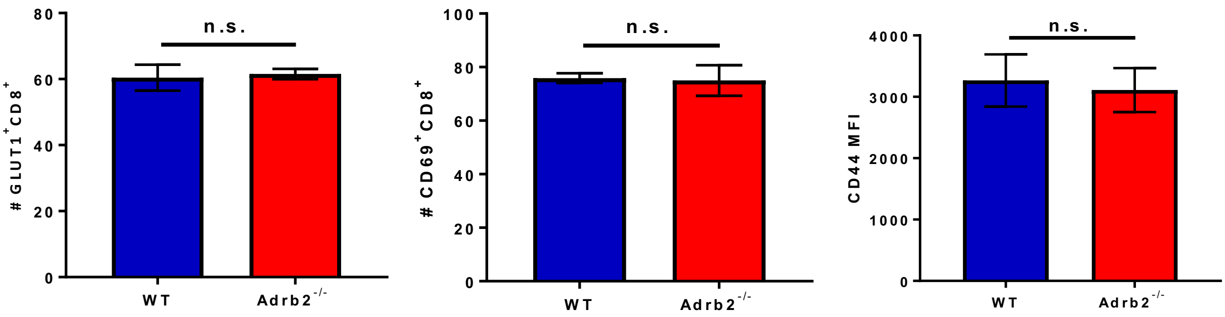

Supplemental Figure 5. There is no difference between CD8$^+$ T-cells cell from wildtype and adrb2$^{-/-}$ mice CD8$^+$ T-cells from BALB/c (wildtype) or adrb2$^{-/-}$ were isolated and purified from lymph node and spleen and activated with anti-CD3/CD28 antibodies Expression of the activation marker CD69, CD44 and GLUT1 were assessed at 24 hours. n=3.Data was analyzed using Student's t test.

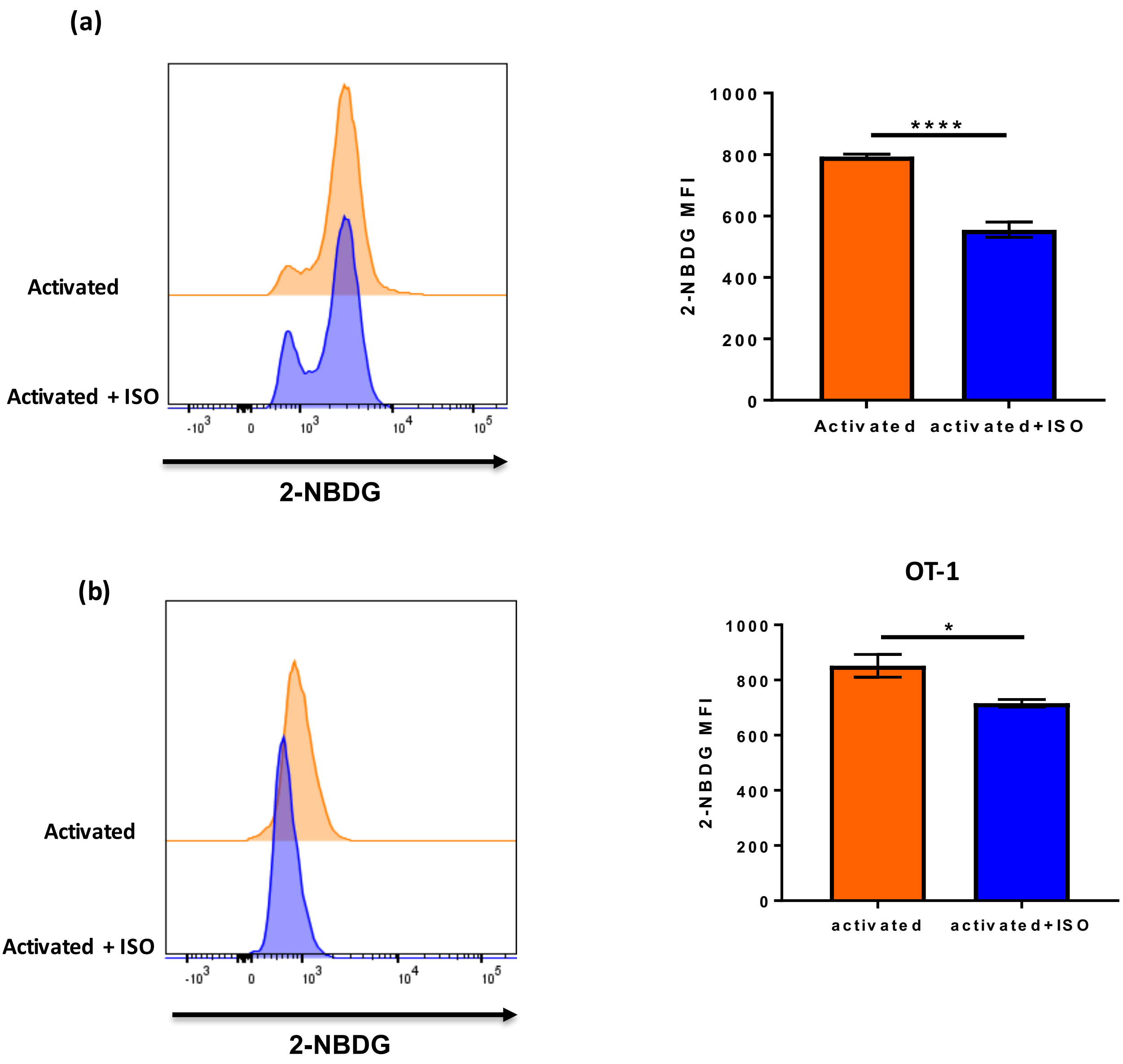

Supplemental Figure 6. β-AR signaling inhibits glucose uptake during T cell activation $CD8^+$ T-cells were isolated from **(a)** C57BL/6 mice or **(b)** OT-1 mice. n=4-6; Data was analyzed using Student's t test , ****p<0.0001.

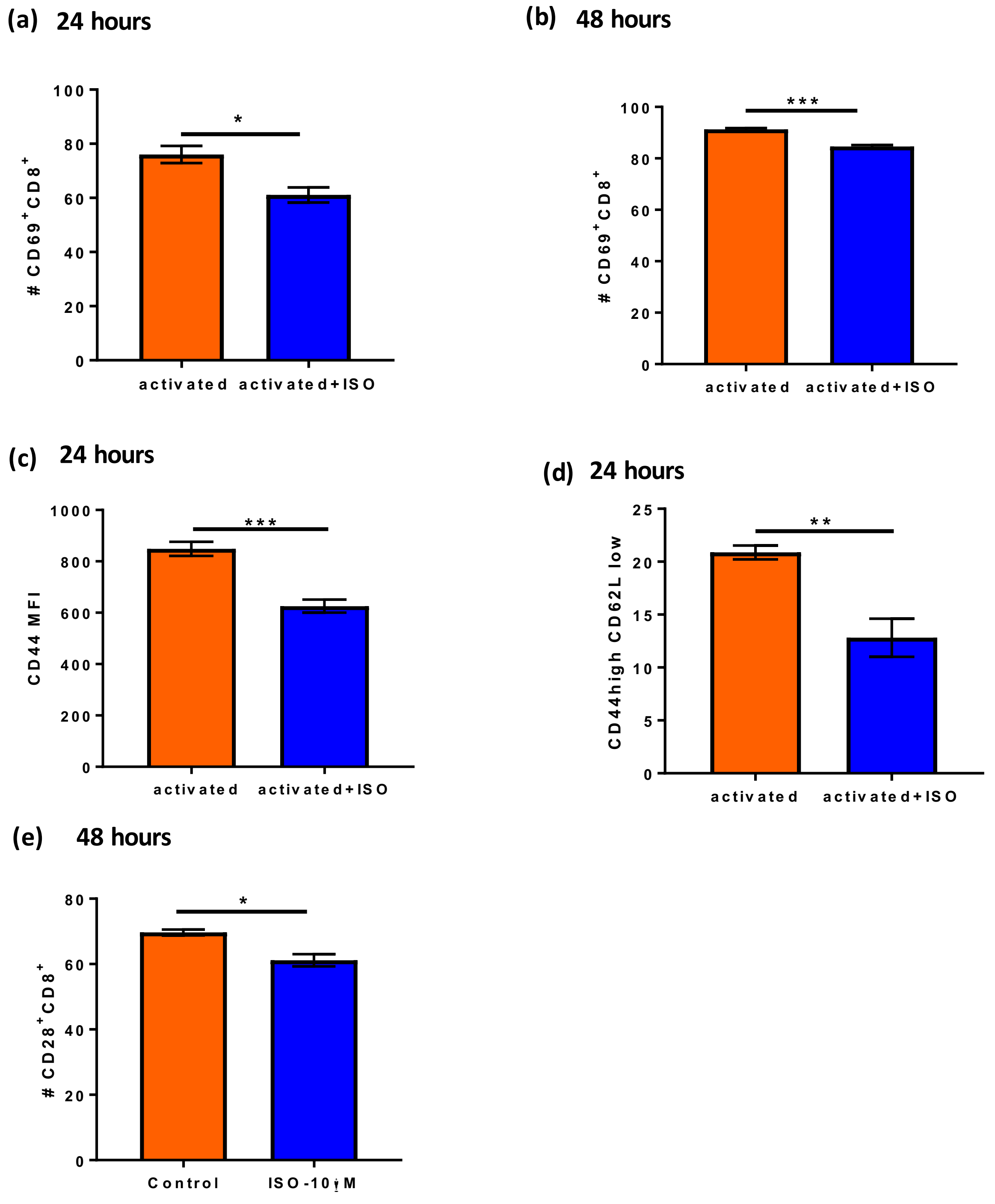

Supplemental Figure 7. β-AR signaling inhibits T cell activation $CD8^+$ T-cells from BALB/c mice were isolated and purified from lymph node and spleen and activated with anti-CD3/CD28 antibodies with or without isoproterenol. **(a, b)** Expression of the activation marker CD69 was assessed at 24 hours **(a)** and 48 hours **(b)** by flow cytometry. **(c, d)** Expression of the activation markers CD44 and CD62L were measured at 24 hours by flow cytometry. **(e)** Expression of the co-stimulatory molecule CD28 was measured at 48 hours by flow cytometry n=3-4.Data was analyzed using Student's t test, $*p<0.05$, $***p<0.001$.

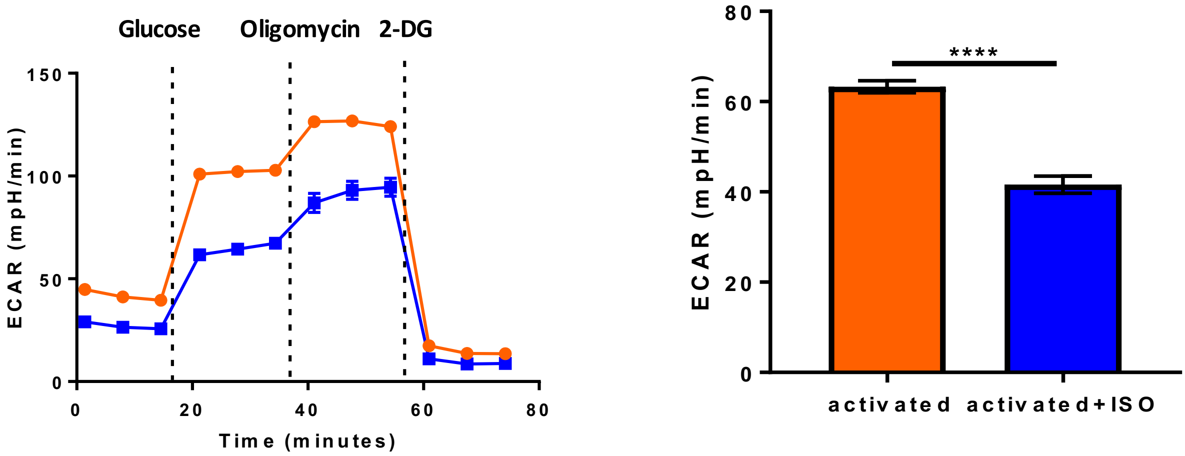

Supplemental Figure 8. β2-AR signaling inhibits glycolysis during T cell activation CD8+ T-cells were isolated from C57BL/6 mice. n=6; Data was analyzed using Student's t test, ****p<0.0001

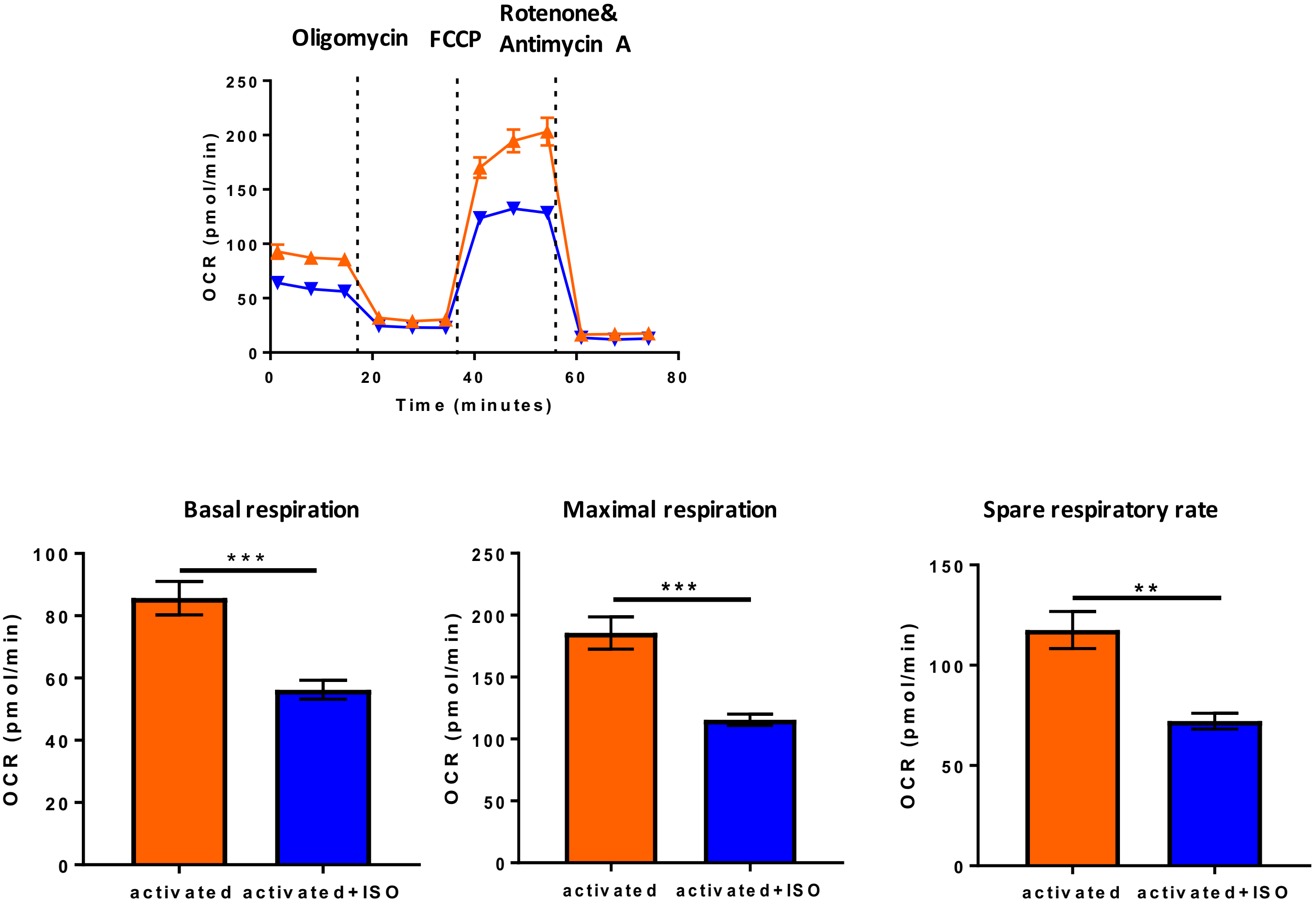

Supplemental Figure 9. β-AR signaling inhibits mitochondrial respiration during T cell activation
CD8^{+} T-cells were isolated from C57BL/6 mice. n=6; Data was analyzed using Student's t test, **$p<0.01$ ***$p<0.001$.
